# Facile Fabrication of *N*-Type Flexible CoSb_3-x_Te_x_ Skutterudite/PEDOT:PSS Hybrid Thermoelectric Films

**DOI:** 10.3390/polym14101986

**Published:** 2022-05-13

**Authors:** Asahi Kato, Cédric Bourgès, Hong Pang, Daniel Gutiérrez, Takeaki Sakurai, Takao Mori

**Affiliations:** 1 International Center for Materials Nanoarchitectonics (WPI-MANA), NIMS, 1-1 Namiki, Tsukuba 305-0044, Ibaraki, Japan; kato.asahi@nims.go.jp (A.K.); bourges.cedric@nims.go.jp (C.B.); gutierez.daniel@nims.go.jp (D.G.); 2 Graduate School of Pure and Applied Sciences, University of Tsukuba, 1-1-1 Tennodai, Tsukuba 305-8577, Ibaraki, Japan

**Keywords:** *n*-type, skutterudite, PEDOT:PSS, film, flexible, thermoelectric

## Abstract

Alongiside the growing demand for wearable and implantable electronics, the development of flexible thermoelectric (FTE) materials holds great promise and has recently become a highly necessitated and efficient method for converting heat to electricity. Conductive polymers were widely used in previous research; however, *n*-type polymers suffer from instability compared to the *p*-type polymers, which results in a deficiency in the *n*-type TE leg for FTE devices. The development of the *n*-type FTE is still at a relatively early stage with limited applicable materials, insufficient conversion efficiency, and issues such as an undesirably high cost or toxic element consumption. In this work, as a prototype, a flexible *n*-type rare-earth free skutterudite (CoSb_3_)/poly(3,4-ethylenedioxythiophene)-polystyrene sulfonate (PEDOT:PSS) binary thermoelectric film was fabricated based on ball-milled skutterudite via a facile top-down method, which is promising to be widely applicable to the hybridization of conventional bulk TE materials. The polymers bridge the separated thermoelectric particles and provide a conducting pathway for carriers, leading to an enhancement in electrical conductivity and a competitive Seebeck coefficient. The current work proposes a rational design towards FTE devices and provides a perspective for the exploration of conventional thermoelectric materials for wearable electronics.

## 1. Introduction

Out of the primary energy sources such as feedstocks, oil, natural gas, etc., there has been an increasing demand for the development of renewable and sustainable sources of energy [1,2,3]. Heat conversion via thermoelectric (TE) devices represents a promising avenue for generating electricity and clean energy in a renewable and sustainable way for future energy development [4,5,6]. Various ongoing efforts in experiments and theories have been attempting to improve the TE properties and conversion efficiency of relevant materials [7,8,9,10]. The efficiency of TE materials is described by a dimensionless figure of merit *ZT* = *S*^2^*σTκ*^−1^, where *S* represents the Seebeck coefficient, *σ* represents electrical conductivity, *κ* represents thermal conductivity, and *T* represents absolute temperature. To improve the figure of merit, the majority of recent research is focused on two aspects: the enhancement of the power factor (*S*^2^*σ*) [11,12,13,14,15,16,17] and the reduction in thermal conductivity *κ* [18,19,20,21,22,23,24,25,26,27].

In recent years, a variety of novel flexible electronic devices, ranging from wearable smart electronics to printable circuit boards, have steadily been developed in line with the concept of the Internet of things (IoT) society [28,29]. The growing demand for wearable and implantable electronics and sensors that use body heat advances the development of flexible thermoelectric (FTE) devices [30,31,32,33,34,35]. Polymers are one of the promising candidates for FTE conversion materials. Most of the advances achieved in FTE materials so far have been focused on conductive polymer-based TE materials. Typical conductive polymers including Polyaniline (PANI) [36], poly(3,4-ethylenedioxythiophene) (PEDOT) [37], Polypyrrole (PPy) [38], etc., show *p*-type TE performance. Benzodifurandione-based polyphenylene vinylene (BDPPV) [39] and poly(nickel-1,1,2,2-ethenetetrathiolate) (poly(Kx(Ni-ett)) [40] as conductive polymers exhibit *n*-type TE performance. However, the *n*-type conductive polymers have a lower TE performance and stability in air compared to *p*-type polymers due to their unstable dopants [33]. Therefore, *p*-type polymers are still the mainstream in current FTE development. Among the various polymers, poly(3,4-ethylenedioxythiophene) polystyrene sulfonate (PEDOT:PSS) stands out in particular because of its high electrical conductivity of up to 4839 S/cm [41], and the possibility of further increasing its TE property via post-treatment with acids or reducing agents [42,43]. Despite its high electrical conductivity and flexibility to be easily tailored into various shapes, the Seebeck coefficient of PEDOT:PSS has remained limited with a value lying in the 10.35–67 µV/K range [41,44], which is far from satisfactory when compared to conventional inorganic TE materials. To overcome the rigidity of the conventional inorganic TE materials and the low performance of polymers, the polymers and inorganic TE materials have been hybridized into an assembly.

Various methods have been conducted to build a hybrid composed of inorganic TE materials and polymers. For example, polyethyleneimine (PEI) has been used as a dopant in carbon nanotube yarn (CNTY) by donating electrons to fabricate an *n*-type FTE film [45]. Furthermore, depositing the specific *n*-type inorganic TE materials on flexible substrates such as nylon and/or PI substrates by suction filtration [46] or magnetron sputtering [47] could also effectively provide flexibility comparable to that of organic materials. However, TE materials that exhibit high performance usually contain relatively expensive and rare elements such as Ag and Te, or toxic elements such as Se. In addition, to produce uniform films, most inorganic materials are synthesized into nanoparticles using chemical processing, but the number of inorganic materials that can be obtained using the same method is limited. Recently, the hybridized FTE films between the ball-milled chalcopyrite (Cu_x_Zn_1-x_FeS_2_) and PEDOT:PSS on the polytetrafluoroethylene (PTFE) membrane have been reported and exhibit outstanding flexibility and performance [48]. This versatile and promising approach to obtaining FTE film could be extended to various inorganic TE materials that present natively attractive TE performance.

Skutterudite, with a general formula of CoSb_3_, is another family of promising TE candidates which shows high TE performance in the medium to high-temperature range due to its high electrical conductivity [49]. By doping CoSb_3_ with Te to form a ternary solid solution alloy, Te provides electrons as a donor, and the electrical conductivity increases rapidly due to the increase in the carrier concentration [50]. Since CoSb_3-x_Te_x_ has been mainly studied as a bulk TE material, the study of CoSb_3-x_Te_x_ as an FTE film will provide new insights and is expected to broaden the selection of inorganic materials used in the development of FTE materials in the future.

In this study, an *n*-type flexible CoSb_3-x_Te_x_/PEDOT:PSS TE film was produced using a facile method. CoSb_3-x_Te_x_ ingots were prepared using a top-down method by breaking down the size of the bulk counterpart via ball-milling that does not require chemical treatment, and the powder was mechanically mixed with PEDOT:PSS, followed by suction filtering onto a membrane. The TE properties (electric conductivity, Seebeck coefficient, power factor, etc.) with different compositions and Te doping of the hybrid films were evaluated and discussed, which demonstrated the potential for CoSb_3-x_Te_x_ to be developed into an FTE component.

## 2. Experimental Section

### 2.1. Materials

Analytical grade Co (pieces, 99.5%) and dimethyl sulfoxide (DMSO) were purchased from Sigma-Aldrich. Analytical grade Sb (grains, 99.9995%) and Te (grains, 99.9995%) were purchased from Kojundo Chemical Laboratory corporation. PEDOT:PSS (1.0–1.3 wt% in water, Clevios PH 1000) was purchased from Heraeus Clevios. Mixed cellulose ester (MCE) membrane filter (with an average pore diameter of ~0.22 µm) was purchased from AS ONE corporation. All reagents were used directly without purification.

### 2.2. Preparation of CoSb_3-x_Te_x_ Skutterudite

CoSb_3-x_Te_x_ (x = 0.05, 0.10, 0.15) was synthesized by a solid-state reaction. Co pieces, Sb grains, and Te grains were well-mixed in a stoichiometric ratio and added into quartz tubes that were subsequently sealed under vacuum conditions (1 × 10^−3^ mbar). The tubes were put into a muffle furnace, which was heated to 1423 K with a ramping rate of 0.8 K/min and kept for 24 h. The samples were quenched in water and annealed at 873 K for 96 h. The as-prepared ingot samples were ground with a mortar to obtain the powder, and the powder was ball-milled for 1 h by the 8000D Mixer/Mill.

### 2.3. Fabrication of the CoSb_3-x_Te_x_ Skutterudite/PEDOT:PSS Composite Films

**Scheme 1:**
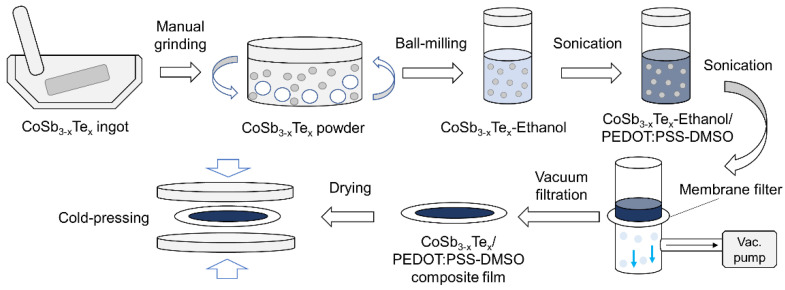
Schematic diagram of the preparation of the CoSb_3-x_Te_x_ skutterudite/PEDOT:PSS film.

The fabrication process for the CoSb_3-x_Te_x_ skutterudite/PEDOT:PSS film is shown in Scheme 1. An aqueous solution of PEDOT:PSS and 5 vol% DMSO was mixed for 2 h using a stirrer, and the mixture was filtered through a polyvinylidene difluoride (PVDF) syringe filter (0.45 µm). Subsequently, the ball-milled CoSb_3-x_Te_x_ powders were added to ethanol and sonicated for 30 min. A CoSb_3-x_Te_x_ dispersion with various skutterudite contents (20, 40, 60, 70, 80, 85, 90, 95, 97, and 98 wt%) was added to the PEDOT:PSS/DMSO solution and sonicated for 30 min. The mixture was vacuum-filtrated with an MCE membrane filter. After the film was dried in a vacuum oven at 60 °C for 10 h, the film was cold-pressed for 5 min at 20MPa. All of the TE properties were measured using MCE membrane-based hybrid films.

### 2.4. Characterization

The morphology and elemental mapping was obtained from a field-emission scanning electron microscope (FE-SEM, Hitachi S-4800, Tokyo, Japan) with an energy-dispersive X-ray spectrometer (EDX, Horiba EMAX Evolution X-Max 80, Kyoto, Japan). In the SEM observation, a small piece of as-prepared film was pasted onto the SEM stage with carbon conductive tape. The accelerating voltage was 10.0 kV. The phase structural information was acquired from the X-ray diffractometer (Smart Lab3, Rigaku Co., Tokyo, Japan) with Cu Kα radiation (λ = 0.154 nm). The measurement was conducted under a voltage of 40 kV and a current of 40 mA (scan speed: 3°/min; scan step: 0.02°) in the Theta-2 Theta geometry. X-ray powder diffraction patterns were refined via Rietveld analysis using the FullProf and WinPLOTR software packages [51,52]. The shape of the diffraction peaks was modeled using a pseudo-Voigt profile function. Zero-point shift, asymmetry parameters, and lattice parameters were systematically refined, and the background contribution was manually estimated. The structure of the organic compounds was characterized by Fourier transform infrared spectroscopy (FT-IR, IRAffinity-1S, Shimadzu, Kyoto, Japan). Thermogravimetric analysis (TGA) was conducted using an STA449 F1 Jupiter (NETZSCH, Selb, Germany) at a heating rate of 10 K/min under a N_2_ atmosphere. The calibration was performed with an empty aluminum crucible. The evaluation of the thermoelectric properties including the electrical conductivity (*σ*), the Seebeck coefficient (*S*), and the power factor (PF) was carried out on a ZEM-3 (Advance Riko, Yokohama, Japan). The measurement was performed in a standard four-probe configuration under a partial helium atmosphere (0.1 MPa) at RT.

## 3. Results and Discussion

**Figure 1:**
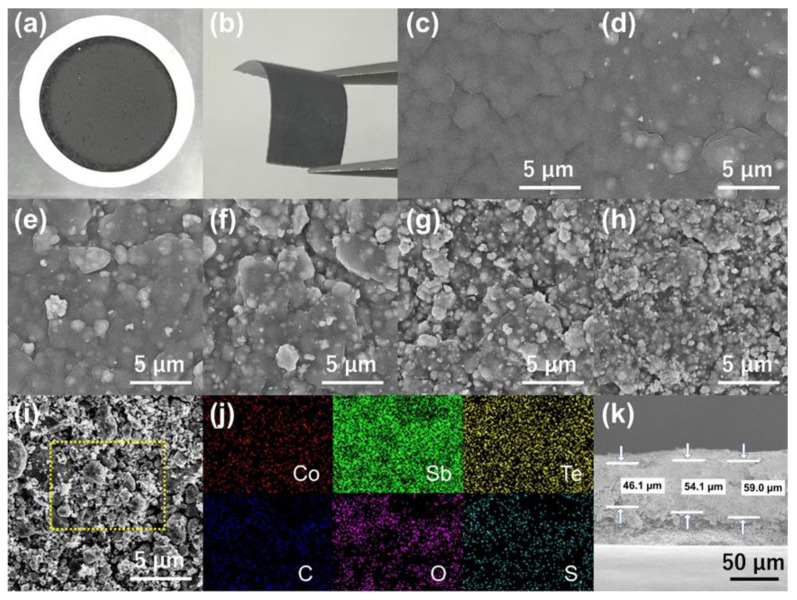
(**a**,**b**) The digital photo images of the 98 wt% CoSb_2.95_Te_0.05_/PEDOT:PSS hybrid film; SEM images of (**c**) pristine PEDOT:PSS-5% DMSO, (**d**) 20 wt%, (**e**) 40 wt%, (**f**) 60 wt%, (**g**) 80 wt%, (**h**) 90 wt% and (**i**) 98 wt% CoSb_2.95_Te_0.05_ hybrid film; (**j**) the corresponding EDX mapping images of Co, Sb, Te, C, O, and S; and (**k**) cross-sectional SEM image of the 98 wt% CoSb_2.95_Te_0.05_ hybrid film.

Figure S1a shows the morphology of the representative as-synthesized CoSb_2.95_Te_0.05_ powder with the corresponding EDX spectrum presenting the elemental Co, Sb, and Te distribution in Figure S1b. The corresponding XRD patterns of the as-synthesized CoSb_3-x_Te_x_ powders with different amounts of Te dopants are shown in Figure S2. The ball-milled powder is consistent with the CoSb_3_ composition, which confirms the purity of the CoSb_2.95_Te_0.05_ prepared in this study. All the patterns can be indexed to the skutterudite structure (JCPDS no.01-083-0055) with a space group of *Im*-3 *(No.204)*. The Rietveld refinement results are summarized in Table S1 and exhibit low-reliability factors demonstrating evidence of how the CoSb_3_ structure purity is exempt from structural defects. Moreover, the lattice size varied linearly with the Te content attesting to the successful substitution of the Sb by the Te within the structure. Figure 1 shows the morphology and microstructural information of the CoSb_2.95_Te_0.05_/PEDOT:PSS hybrid film. According to the digital photo image of the 98 wt% CoSb_2.95_Te_0.05_/PEDOT:PSS hybrid film shown in Figure 1a, the color of the hybrid film is black, similar to the color of CoSb_2.95_Te_0.05_ powder. As displayed in Figure 1b, the hybrid film retains its flexibility without obvious cracks in the bent state even though the content of the CoSb_2.95_Te_0.05_ powder is very high. The morphology of the hybrid CoSb_2.95_Te_0.05_/PEDOT:PSS films with different fractions of PEDOT:PSS can be found in the SEM images in Figure 1c–i, from which it is noteworthy that the PEDOT:PSS significantly affects the homogeneity of the hybrid film. The contours of the CoSb_2.95_Te_0.05_ particles become more obvious with the increase in the weight percentage of the inorganic components. From the top view image of the 98 wt% CoSb_2.95_Te_0.05_/PEDOT:PSS hybrid film shown in Figure 1i, CoSb_2.95_Te_0.05_ particles can be observed with sizes ranging from submicron to several microns spread on the surface of the hybrid film. In the EDX mapping (Figure 1j) corresponding to the SEM image shown in Figure 1i, the uniform distribution of the elements Co, Sb, and Te, which constitute CoSb_2.95_Te_0.05_, and the C, O, and S contained in PEDOT:PSS reveal how the TE materials and PEDOT:PSS are evenly dispersed within each other. The cross-sectional image in Figure 1k indicates that the typical film in our experiment has a thickness of 53.0 ± 7 μm.

**Figure 2:**
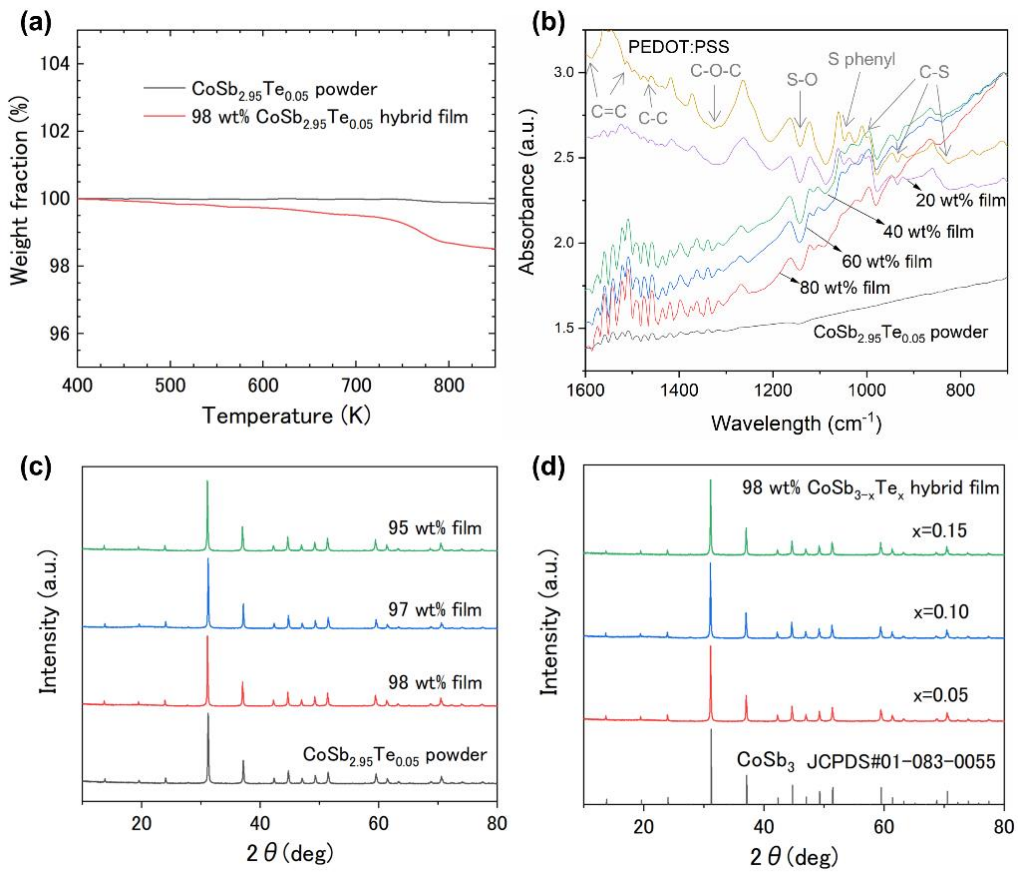
(**a**) TGA data of the 98 wt% CoSb_2.95_Te_0.05_ hybrid film and the CoSb_2.95_Te_0.05_ powder; (**b**) FT-IR spectra of the pristine PEDOT:PSS film, the CoSb_2.95_Te_0.05_ hybrid film (20, 40, 60, and 80 wt%), and the CoSb_2.95_Te_0.05_ powder; (**c**) XRD patterns of the CoSb_2.95_Te_0.05_ hybrid film (95, 97, and 98 wt%) and the CoSb_2.95_Te_0.05_ powder; (**d**) XRD patterns of the 98 wt% CoSb_3-x_Te_x_ hybrid film with different amount of Te dopants (x = 0.05, 0.10, and 0.15).

According to the TGA results shown in Figure 2a, even in the hybrid film with 98 wt% CoSb_2.95_Te_0.05_ where the ratio of the PEDOT:PSS is extremely small, it is evident that a mass loss occurrs corresponding to the decomposition of the PEDOT:PSS when increasing the measurement temperature, confirming the successful assembly of the PEDOT:PSS and the CoSb_2.95_Te_0.05_ powders_._ Figure 2b shows the FT-IR spectra of the CoSb_2.95_Te_0.05_/PEDOT:PSS hybrid films. It is known that PEDOT:PSS has several distinct absorption bands between 800 cm^−1^ and 1600 cm^−1^ [53,54], and a similar spectrum was observed for the PEDOT:PSS used in this study. As the amount of CoSb_2.95_Te_0.05_ increases, the signature peaks of PEDOT:PSS are weakened, but the peak position of C-S at wavenumber 977 cm^−1^ and S-O at wavenumber 1141 cm^−1^ are not shifted, indicating that the structure of the PEDOT:PSS has not changed.

**Figure 3:**
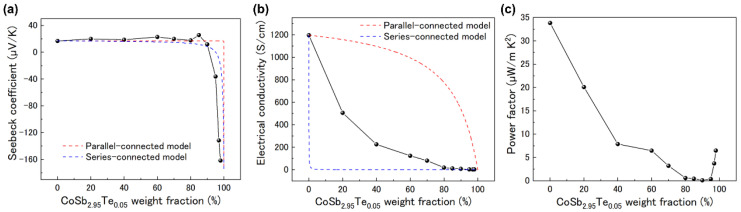
Thermoelectric properties of the hybrid films at room temperature as a function of the weight percentage (0, 20, 40, 60, 70, 80, 85, 90, 95, 97, 98, and 100 wt%) of the CoSb_2.95_Te_0.05_ in the hybrid films with PEDOT:PSS: (**a**) Seebeck coefficient, (**b**) electrical conductivity, and (**c**) power factor.

The properties of the hybrid films in Figure 3 containing a lower content (<95 wt%) of CoSb_2.95_Te_0.05_ show much lower TE properties than the hybrid films containing a CoSb_2.95_Te_0.05_ content above 95 wt%. Our experiments in the later section are mainly focused on the hybrid films with the CoSb_3-x_Te_x_ weight fraction over 95 wt%. The XRD patterns of the hybrid films with 95 wt%, 97 wt%, and 98 wt% of CoSb_2.95_Te_0.05_ are shown here in Figure 2c, which are very consistent with the XRD patterns of the original powders in Figure S2. The structure of CoSb_2.95_Te_0.05_ did not change, indicating that no mutual interference on the microstructure occurred between the CoSb_2.95_Te_0.05_ and the PEDOT:PSS during the hybridization of the two materials. The lattice sizes of all the CoSb_2.95_Te_0.05_ films are comparable to the reference powder, attesting to the non-degradation of the powder during the hybridization with PEDOT:PSS. The same trend was also observed when the doping concentration of CoSb_3-x_Te_x_ in the hybrid film was varied (Figure 2d).

The TE properties of the hybrid films were first investigated containing various weight fractions of CoSb_2.95_Te_0.05_. As shown in Figure 3, there was no significant difference in the Seebeck coefficient of the hybrid films with CoSb_3-x_Te_x_ percentages ranging from 0 wt% to 90 wt% except for a small fluctuation at 80 wt%. The positive signs of the Seebeck coefficient of the CoSb_2.95_Te_0.05_/PEDOT:PSS hybrid film showed *p*-type features until the content of CoSb_2.95_Te_0.05_ increased up to 90 wt%, attesting to the dominant properties of the PEDOT:PSS. The hybrid film started to show the *n*-type features with the CoSb_2.95_Te_0.05_ weight fraction more than 95 wt%. The Seebeck coefficient of the hybrid film is a compromise of the *n*-type property of CoSb_3-x_Te_x_ and the *p*-type property of PEDOT:PSS. Thus, with an excessive amount of PEDOT:PSS, the dominant charge carriers in the hybrid film are holes. In order to fabricate the *n*-type film, it is necessary to increase the fraction of skutterudite to above 95 wt%. The electrical conductivity decreases gradually while increasing the amount of CoSb_3-x_Te_x_ powder. In comparison, the CoSb_2.95_Te_0.05_ film was also fabricated using the same method for the hybrid film without the addition of PEDOT:PSS, which only showed 0.031 S/cm as displayed in Table S2. The very low electrical conductivity can be attributed to the skutterudite particles aggregating in a random way to form a loose structure on the film where there is no effective bonding between the CoSb_2.95_Te_0.05_ particles (Figure S3). While improving the amount of PEDOT:PSS to bridge the particles, the electrical conductivity shows a drastic upward trend. According to the previous method [55], we analyzed the composite method between CoSb_2.95_Te_0.05_ and PEDOT:PSS. As shown in Figure 3a, the red line is fitted in a parallel-connected model with *S* and *σ* as in the follow the Equation:

(1) $$S_{parallel}=\frac{x_{s}\sigma_{s}S_{s}+\left(1-x_{s}\right)\sigma_{p}S_{p}}{x_{s}\sigma_{s}+\left(1-x_{s}\right)\sigma_{p}}$$

(2) $$\sigma_{parallel}=x_{s}\sigma_{s}+\left(1-x_{s}\right)\sigma_{p}$$

where *S_parallel_* and *σ_parallel_* are the Seebeck coefficient and the electrical conductivity of the parallel-connected composite; *S_s_* and *S_p_* are the Seebeck coefficients of CoSb_2.95_Te_0.05_ and PEDOT:PSS, respectively; *σ_s_* and *σ_p_* are the electrical conductivities of CoSb_2.95_Te_0.05_ and PEDOT:PSS, respectively; and *x_s_* is the volume fraction of CoSb_2.95_Te_0.05_. 

The blue line is fitted in a series-connected model with *S* and *σ* as in the following equation:

(3) $$S_{series}=\frac{x_{s}\kappa_{p}S_{s}+\left(1-x_{s}\right)\kappa_{s}S_{p}}{x_{s}\kappa_{p}+\left(1-x_{s}\right)\kappa_{s}}$$

(4) $$\sigma_{series}{}^{-1}=x_{s}\sigma_{s}{}^{-1}+\left(1-x_{s}\right)\sigma_{p}{}^{-1}$$

where *S_series_* and *σ_series_* are the Seebeck coefficient and the electrical conductivity of the series-connected composite, and *κ_s_* and *κ_p_* are the thermal conductivities of CoSb_2.95_Te_0.05_ and PEDOT:PSS, respectively. The experimental values of the Seebeck coefficient and electrical conductivity show properties closer to those of the series-connected model.

**Figure 4:**
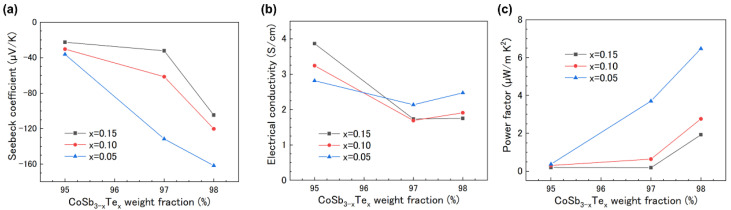
Thermoelectric properties of the hybrid films at room temperature as a function of the weight percentage (95, 97, and 98 wt%) of the CoSb_3-x_Te_x_ (x = 0.05, 0.10, and 0.15) in the hybrid films with PEDOT:PSS: (**a**) Seebeck coefficient, (**b**) electrical conductivity, and (**c**) power factor.

To optimize the CoSb_3_-based FTE film, the doping level of the native inorganic powder was modulated to promote a larger negative Seebeck coefficient. Different doping ratios of CoSb_3-x_Te_x_ (x = 0.05, 0.10, 0.15) and of skutterudite hybridized with different mass ratios of X CoSb_3-x_Te_x_/1-X PEDOT:PSS (X = 95, 97, 98 wt%) have been developed, and the influence on the FTE properties was investigated as shown in Figure 4. With the same fraction of skutterudite, the Seebeck coefficient of the hybrid film increases with a decreasing CoSb_3-x_Te_x_ doping ratio and obtained a largest negative value of −161.7 μV/K at 98 wt%, considering an uncertainty of 6% from the measurement [56]. This trend is also observed in the CoSb_3-x_Te_x_ bulk sample, where the Seebeck coefficient increases with a decreasing doping rate due to the charge carrier tuning (Table S3). In other words, a larger Seebeck coefficient in the native powder likely helps to reach a higher Seebeck coefficient in the hybrid FTE film. The contribution of CoSb_3-x_Te_x_ to the Seebeck coefficient is dominant in the *n*-type film with a high CoSb_3-x_Te_x_ content. However, there is only a small variation in the electrical conductivity of the hybrid film when decreasing the CoSb_3-x_Te_x_ doping ratio, and the electrical conductivity generates a different trend with the variation in the Te doping ratio in the CoSb_3-x_Te_x_. The skutterudite grains of micrometer size synthesized under high vacuum are very stable with a negligible effect from the surface oxidation; this is thought to be due to the electrical conduction of the hybrid film being modulated by the interaction of the PEDOT:PSS and the bulk CoSb_3-x_Te_x_ particles. The electrical conductivity at various doping levels of Te showed a decreasing trend when the weight fraction increased from 90 wt% to 97 wt%. However, at 98 wt%, the electrical conductivity slightly increased compared to the 97 wt%, which might be due to the slightly larger compacity of CoSb_3-x_Te_x_ powders within the film. In addition, we compared the electrical conductivity with different doping levels at the same weight fraction. The CoSb_2.85_Te_0.15_/PEDOT:PSS film shows a much higher electrical conductivity than the CoSb_2.90_Te_0.10_/PEDOT:PSS and CoSb_2.95_Te_0.05_/PEDOT:PSS at 95 wt%, but it becomes the lowest at 98 wt%. At the same time, CoSb_2.95_Te_0.05_/PEDOT:PSS becomes the best hybrid film when improving the weight fraction above 97 wt%, while CoSb_2.85_Te_0.15_/PEDOT:PSS and CoSb_2.90_Te_0.10_/PEDOT:PSS generate similar electrical conductivities. The exact mechanism is quite complex but might be related to the drastic decrease of the carrier mobility with doped Te incremented as indicated previously in the reference. As a result, the hybrid film with the smallest doping ratio (x = 0.05) of Te, CoSb_2.95_Te_0.05_, at 98 wt% shows the largest power factor of 6.47 µW/m K^2^ at room temperature.

Table 1 shows the Seebeck coefficient, electrical conductivity, and power factor of the *n*-type flexible thermoelectric materials reported so far. In this study, CoSb_2.95_Te_0.05_/PEDOT:PSS hybrid film records the largest negative Seebeck coefficient value of −161.7 µV/K at ambient temperature. However, the electrical conductivity and the power factor are much lower compared to the previous work. We will improve our efforts in the future by downsizing the skutterudites and uniformizing the grain size. In addition, we measured the temperature-dependent TE properties of the 98wt% CoSb_2.95_Te_0.05_ hybrid film as shown in Figure S4. Both the electrical conductivity and the absolute value of Seebeck coefficient increased with an increase in temperature. This is a typical metallic behavior dependence observed in Te-doped CoSb_3_, which agrees with a large carrier concentration and supports the idea that the inorganic component led the electrical transport properties rather than the organic component.

**Figure 5:**
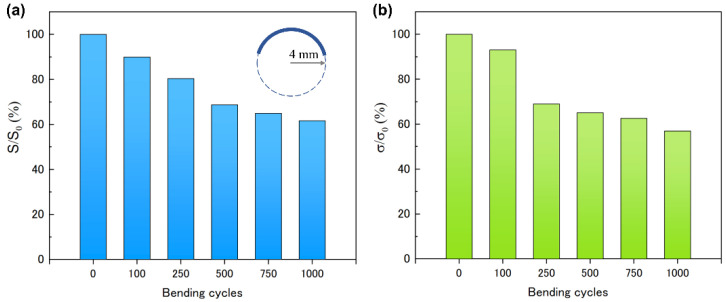
(**a**) Seebeck coefficient and (**b**) electrical conductivity of the 98 wt% CoSb_2.95_Te_0.05_ hybrid film as a function of bending cycles.

The 98 wt% CoSb_2.95_Te_0.05_/PEDOT:PSS hybrid film, which has the highest thermoelectric performance, was subjected to a bending test to measure its flexibility as shown in Figure 5. The Seebeck coefficient and the electrical conductivity decreased from the original value promptly in the first 250 bending cycles along a glass rod with a 4 mm radius, but the rate of decrease became much slower after 250 bending cycles, demonstrating the film’s flexibility to a certain degree considering that the film contains a large weight of powder and the facile method for breaking down the size of rigid TE materials.

## 4. Conclusions

In this study, a CoSb_3-x_Te_x_/PEDOT:PSS hybrid film was produced using a simple method. First, we succeeded in our preliminary step to obtain a submicron to several micron particle size suitable for hybridization with the organic counterpart. Then, by suction-filtering the mixed solution with the PEDOT:PSS aqueous dispersion to an MCE membrane, we achieved the production of a uniform and flexible *n*-type FTE film. As for the TE performance, the Seebeck coefficient of the hybrid film increased as the amount of CoSb_3-x_Te_x_ in the hybrid film increased and the doping rate of CoSb_3-x_Te_x_ decreased. As a result, the CoSb_2.95_Te_0.05_/PEDOT:PSS hybrid film obtained the largest negative Seebeck coefficient of −161.7 µV/K at 98 wt% at room temperature. As it is more challenging to obtain a large negative Seebeck coefficient in the hybrid thin films than improving the electrical conductivity of these type of films, wherein few approaches have been attempted, our work established a useful method of improving the power factor by raising the Seebeck coefficient. In addition, the 98 wt% hybrid film retained the flexibility to maintain a certain degree of electrical conductivity even after being bent 1000 times. It was proved that it is relatively easy to fabricate a flexible film with a very small amount of PEDOT:PSS and obtain moderate properties by finely tuning the composition of the TE films. The same method in this study can be applied to other inorganic materials. Therefore, it is expected that the selection of inorganic TE materials used for the development of *n*-type flexible thermoelectric materials will be expanded in the future.

## Figures and Tables

**Table 1:** Comparison of the reported Seebeck coefficient of the *n*-type flexible TE devices at room temperature.

TE Materials	Seebeck Coefficient (µV/K)	Conductivity (S/cm)	Power Factor (µW/m K^2^)	Reference
SWCNT/PEI	−56	7850	2456	[45]
C_60_/TiS_2_	−101	390	375	[57]
PEDOT/Ag_2_Se /CuAgSe	−121.8	1200	1603	[58]
PEDOT:PSS/Bi_2_Te_3_	−137.8	73	138.6	[59]
CoSb_2.95_Te_0.05_ /PEDOT:PSS	−161.7	2.47	6.46	This work

## Data Availability

All the experimental data obtained are presented, in the form of figures or/and tables, in the article and in the Supplementary Information, and are available from the corresponding author upon reasonable request.

## Supplementary Materials

The following supporting information can be downloaded at https://www.mdpi.com/article/10.3390/polym14101986/s1, Figure S1: (a) SEM image and EDX mapping images of the ball-milled CoSb_2.95_Te_0.05_ powder; (b) the corresponding EDX spectrum; Figure S2: XRD patterns of the ball-milled CoSb_3-x_Te_x_ powder with different amounts of Te dopants (x = 0.05, 0.10, 0.15); Table S1: Cell parameters and reliability factors obtained from Rietveld refinement of XRD patterns (λ_Cu_ = 1.54056 Å) of the CoSb_3-x_Te_x_ ball-milled powders and X CoSb_2.95_Te_0.05_/1-X PEDOT:PSS hybrid films; Table S2: Thermoelectric properties of PEDOT:PSS with 5 vol% of DMSO and the CoSb_2.95_Te_0.05_ skutterudite film sample at room temperature; Figure S3: Cold-pressed CoSb_2.95_Te_0.05_ powder film: (a) digital photo image; (b) SEM image; Table S3: Thermoelectric properties of the CoSb_3-x_Te_x_ (x = 0.05, 0.10, 0.15) bulk sample at room temperature. Figure S4: Electrical conductivity and the Seebeck coefficient of the 98 wt% CoSb_2.95_Te_0.05_ hybrid film with increasing temperature [60].

## References

[1] Gunnarsdottir I., Davidsdottir B., Worrell E., Sigurgeirsdottir S. (2021). Sustainable energy development: History of the concept and emerging themes. Renew. Sustain. Energy Rev..

[2] Ikram M. (2021). Models for Predicting Non-Renewable Energy Competing with Renewable Source for Sustainable Energy Development: Case of Asia and Oceania Region. Glob. J. Flex. Syst. Manag..

[3] Siwal S.S., Zhang Q., Devi N., Saini A.K., Saini V., Pareek B., Gaidukovs S., Thakur V.K. (2021). Recovery processes of sustainable energy using different biomass and wastes. Renew. Sustain. Energy Rev..

[4] He J., Tritt T.M. (2017). Advances in thermoelectric materials research: Looking back and moving forward. Science.

[5] Bell L.E. (2008). Cooling, Heating, Generating Power, and Recovering Waste Heat with Thermoelectric Systems. Science.

[6] Liu Z., Sato N., Gao W., Yubuta K., Kawamoto N., Mitome M., Kurashima K., Owada Y., Nagase K., Lee C. (2021). Demonstration of ultrahigh thermoelectric efficiency of 7.3 % in Mg_3_Sb_2_/MgAgSb module for low-temperature energy harvesting. Joule.

[7] Shi X., Zou J., Chen Z.-G. (2020). Advanced Thermoelectric Design: From Materials and Structures to Devices. Chem. Rev..

[8] Snyder G.J., Toberer E.S. (2008). Complex thermoelectric materials. Nat. Mater..

[9] Mori T. (2017). Novel Principles and Nanostructuring Methods for Enhanced Thermoelectrics. Small.

[10] Mao J., Liu Z., Zhou J., Zhu H., Zhang Q., Chen G., Ren Z. (2018). Advances in thermoelectrics. Adv. Phys..

[11] Zebarjadi M., Joshi G., Zhu G., Yu B., Minnich A., Lan Y., Wang X., Dresselhaus M., Ren Z., Chen G. (2011). Power Factor Enhancement by Modulation Doping in Bulk Nanocomposites. Nano Lett..

[12] Watson B.W., Meng L., Fetrow C., Qin Y. (2016). Core/Shell Conjugated Polymer/Quantum Dot Composite Nanofibers through Orthogonal Non-Covalent Interactions. Polymers.

[13] Tamaki H., Sato H.K., Kanno T. (2016). Isotropic Conduction Network and Defect Chemistry in Mg_3+δ_Sb_2_-Based Layered Zintl Compounds with High Thermoelectric Performance. Adv. Mater..

[14] Yang Y., Deng H., Fu Q. (2020). Recent progress on PEDOT:PSS based polymer blends and composites for flexible electronics and thermoelectric devices. Mater. Chem. Front..

[15] Zianni X. (2019). The annealed-nanograin phase: A route to simultaneous increase of the conductivity and the Seebeck coefficient and high thermoelectric performance. J. Appl. Phys..

[16] Bailey T., Lu R., Poudeu P., Uher C. (2019). Mictomagnetic full-Heusler nanoprecipitates in (Ti, Zr, Hf)NiFexSn half-Heusler composites. Mater. Today Phys..

[17] Liu Z., Gao W., Zhang W., Sato N., Guo Q., Mori T. (2020). High Power Factor and Enhanced Thermoelectric Performance in Sc and Bi Codoped GeTe: Insights into the Hidden Role of Rhombohedral Distortion Degree. Adv. Energy Mater..

[18] Khan A.U., Kobayashi K., Tang D., Yamauchi Y., Hasegawa K., Mitome M., Xue Y., Jiang B., Tsuchiya K., Golberg D. (2017). Nano Energy Nano-micro-porous skutterudites with 100 % enhancement in ZT for high performance thermoelectricity. Nano Energy.

[19] Lee S., Esfarjani K., Luo T., Zhou J., Tian Z., Chen G. (2014). Resonant bonding leads to low lattice thermal conductivity. Nat. Commun..

[20] Du B., Chen K., Yan H., Reece M.J. (2016). Efficacy of lone-pair electrons to engender ultralow thermal conductivity. Scr. Mater..

[21] Miyazaki Y., Hamada H., Nagai H., Hayashi K. (2018). Crystal Structure and Thermoelectric Properties of Lightly Substituted Higher Manganese Silicides. Materials.

[22] Roychowdhury S., Jana M.K., Pan J., Guin S.N., Sanyal D., Waghmare U.V., Biswas K. (2018). Soft Phonon Modes Leading to Ultralow Thermal Conductivity and High Thermoelectric Performance in AgCuTe. Angew. Chem..

[23] Zapata-Arteaga O., Perevedentsev A., Marina S., Martin J., Reparaz J.S., Campoy-Quiles M. (2020). Reduction of the Lattice Thermal Conductivity of Polymer Semiconductors by Molecular Doping. ACS Energy Lett..

[24] Long S.O., Powell A.V., Hull S., Orlandi F., Tang C.C., Supka A.R., Fornari M., Vaqueiro P. (2020). Jahn–Teller Driven Electronic Instability in Thermoelectric Tetrahedrite. Adv. Funct. Mater..

[25] Liu Z., Zhang W., Gao W., Mori T. (2021). A material catalogue with glass-like thermal conductivity mediated by crystallographic occupancy for thermoelectric application. Energy Environ. Sci..

[26] Zhu Y., Han Z., Jiang F., Dong E., Zhang B.-P., Zhang W., Liu W. (2021). Thermodynamic criterions of the thermoelectric performance enhancement in Mg_2_Sn through the self-compensation vacancy. Mater. Today Phys..

[27] Li G., He J., An Q., Morozov S.I., Hao S., Zhai P., Zhang Q., Goddard W.A., Snyder G.J. (2019). Dramatically reduced lattice thermal conductivity of Mg_2_Si thermoelectric material from nanotwinning. Acta Mater..

[28] Proto A., Penhaker M., Conforto S., Schmid M. (2017). Nanogenerators for Human Body Energy Harvesting. Trends Biotechnol..

[29] Haras M., Skotnicki T. (2018). Thermoelectricity for IoT—A review. Nano Energy.

[30] Bharti M., Singh A., Samanta S., Aswal D.K. (2017). Conductive polymers: Creating their niche in thermoelectric domain. Prog. Mater. Sci..

[31] Yao C.-J., Zhang H.-L., Zhang Q. (2019). Recent Progress in Thermoelectric Materials Based on Conjugated Polymers. Polymers.

[32] Zhang Y., Park S.-J. (2019). Flexible Organic Thermoelectric Materials and Devices for Wearable Green Energy Harvesting. Polymers.

[33] Masoumi S., O’Shaughnessy S., Pakdel A. (2022). Organic-based flexible thermoelectric generators: From materials to devices. Nano Energy.

[34] Nandihalli N., Liu C.-J., Mori T. (2020). Polymer based thermoelectric nanocomposite materials and devices: Fabrication and characteristics. Nano Energy.

[35] Petsagkourakis I., Tybrandt K., Crispin X., Ohkubo I., Satoh N., Mori T. (2018). Thermoelectric materials and applications for energy harvesting power generation. Sci. Technol. Adv. Mater..

[36] Toshima N., Ichikawa S. (2015). Conducting Polymers and Their Hybrids as Organic Thermoelectric Materials. J. Electron. Mater..

[37] Gueye M.N., Carella A., Faure-Vincent J., Demadrille R., Simonato J.-P. (2020). Progress in understanding structure and transport properties of PEDOT-based materials: A critical review. Prog. Mater. Sci..

[38] Du Y., Niu H., Li J., Dou Y., Shen S.Z., Jia R., Xu J. (2018). Morphologies Tuning of Polypyrrole and Thermoelectric Properties of Polypyrrole Nanowire/Graphene Composites. Polymers.

[39] Shi K., Zhang F., Di C., Yan T., Zou Y., Zhou X., Zhu D., Wang J., Pei J. (2015). Toward High Performance *n*-Type Thermoelectric Materials by Rational Modi fi cation of BDPPV Backbones. J. Am. Chem. Soc..

[40] Sun Y., Sheng P., Di C.-A., Jiao F., Xu W., Qiu D., Zhu D. (2012). Organic Thermoelectric Materials and Devices Based on *p*- and *n*-Type Poly(metal 1,1,2,2-ethenetetrathiolate)s. Adv. Mater..

[41] Bae E.J., Kang Y.H., Jang K.-S., Cho S.Y. (2016). Enhancement of Thermoelectric Properties of PEDOT:PSS and Tellurium-PEDOT:PSS Hybrid Composites by Simple Chemical Treatment. Sci. Rep..

[42] Fan Z., Li P., Du D., Ouyang J. (2017). Significantly Enhanced Thermoelectric Properties of PEDOT:PSS Films through Sequential Post-Treatments with Common Acids and Bases. Adv. Energy Mater..

[43] Yemata T.A., Zheng Y., Kyaw A.K.K., Wang X., Song J., Chin W.S., Xu J. (2020). Modulation of the doping level of PEDOT:PSS film by treatment with hydrazine to improve the Seebeck coefficient. RSC Adv..

[44] Park H., Lee S.H., Kim F.S., Choi H.H., Cheong I.W., Kim J.H. (2014). Enhanced thermoelectric properties of PEDOT:PSS nanofilms by a chemical dedoping process. J. Mater. Chem. A.

[45] Choi J., Jung Y., Yang S.J., Oh J.Y., Oh J., Jo K., Son J.G., Moon S.E., Park C.R., Kim H. (2017). Flexible and Robust Thermoelectric Generators Based on All-Carbon Nanotube Yarn without Metal Electrodes. ACS Nano.

[46] Jiang C., Ding Y., Cai K., Tong L., Lu Y., Zhao W., Wei P. (2020). Ultrahigh Performance of *n*-Type Ag_2_Se Films for Flexible Thermoelectric Power Generators. ACS Appl. Mater. Interfaces.

[47] Kong D., Zhu W., Guo Z., Deng Y. (2019). High-performance flexible Bi_2_Te_3_ films based wearable thermoelectric generator for energy harvesting. Energy.

[48] Wang Y., Pang H., Guo Q., Tsujii N., Baba T., Baba T., Mori T. (2021). Flexible *n*-Type Abundant Chalcopyrite/PEDOT:PSS/Graphene Hybrid Film for Thermoelectric Device Utilizing Low-Grade Heat. ACS Appl. Mater. Interfaces.

[49] Liu Z.-Y., Zhu J.-L., Tong X., Niu S., Zhao W.-Y. (2020). A review of CoSb_3_-based skutterudite thermoelectric materials. J. Adv. Ceram..

[50] Liu W.-S., Zhang B.-P., Li J.-F., Zhang H.-L., Zhao L.-D. (2007). Enhanced thermoelectric properties in CoSb_3-x_Te_x_ alloys prepared by mechanical alloying and spark plasma sintering. J. Appl. Phys..

[51] Rodríguez-Carvajal J. (1993). Recent advances in magnetic structure determination by neutron powder diffraction. Phys. B Condens. Matter.

[52] Roisnel T., Rodríguez-Carvajal J. (2001). WinPLOTR, a graphic tool for powder diffraction. Mater. Sci. Forum.

[53] Xu S., Liu C., Xiao Z., Zhong W., Luo Y., Ou H., Wiezorek J. (2017). Cooperative effect of carbon black and dimethyl sulfoxide on PEDOT:PSS hole transport layer for inverted planar perovskite solar cells. Sol. Energy.

[54] Wang C., Zhang C., Using C.M.T., Electrodes C.P., Yang J. (2018). Effect of localized surface plasmon resonance from incorporated gold nanoparticles in PEDOT:PSS hole transport layer for hybrid solar cell applications. J. Phys..

[55] Park D., Kim M., Kim J. (2020). Fabrication of PEDOT:PSS/Ag_2_Se Nanowires for Polymer-Based Thermoelectric Applications. Polymers.

[56] Alleno E., Bérardan D., Byl C., Candolfi C., Daou R., Decourt R., Guilmeau E., Hébert S., Hejtmanek J., Lenoir B. (2015). A round robin test of the uncertainty on the measurement of the thermoelectric dimensionless figure of merit of Co_0.97_Ni_0.03_Sb_3_. Rev. Sci. Instrum..

[57] Wang L., Zhang Z., Geng L., Yuan T., Liu Y., Guo J., Fang L., Qiu J., Wang S. (2018). Solution-printable fullerene/TiS_2_ organic/inorganic hybrids for high-performance flexible n-type thermoelectrics. Energy Environ. Sci..

[58] Lu Y., Qiu Y., Cai K., Li X., Gao M., Jiang C., He J. (2020). Ultrahigh performance PEDOT/Ag_2_Se/CuAgSe composite film for wearable thermoelectric power generators. Mater. Today Phys..

[59] We J.H., Kim S.J., Cho B.J. (2014). Hybrid composite of screen-printed inorganic thermoelectric film and organic conducting polymer for flexible thermoelectric power generator. Energy.

[60] Li Z., Sun H., Hsiao C., Yao Y., Xiao Y., Shahi M., Jin Y., Cruce A., Liu X., Jiang Y. (2018). A Free-Standing High-Output Power Density Thermoelectric Device Based on Structure-Ordered PEDOT:PSS. Adv. Electron. Mater..

