# Supplementary material for: Facile Fabrication of N-Type Flexible CoSb3-xTex Skutterudite/PEDOT:PSS Hybrid Thermoelectric Films
Source: Polymers (Basel). 2022 May 13;14(10):1986. doi: 10.3390/polym14101986 (PMC9144647; doi:10.3390/polym14101986)
Supplement: Supplementary file 1 [file polymers-14-01986-s001.zip › polymers-1684272-supplementary.pdf]

Supplementary Information

Facile fabrication of *n*-type flexible $CoSb_{3-x}Te_x$ skutterudite/PEDOT:PSS hybrid thermoelectric films

Asahi Kato [1,2], Cédric Bourgès [1], Hong Pang [1], Daniel Gutiérrez [1,2], Takeaki Sakurai [2] and Takao Mori [1,2,*]

[1] International Center for Materials Nanoarchitectonics (WPI-MANA), NIMS, 1-1 Namiki, Tsukuba, Ibaraki, 305-0044, Japan; KATO.Asahi@nims.go.jp (A.K.); PANG.Hong@nims.go.jp (H.P.); BOURGES.Cedric@nims.go.jp (C.B.); MORI.Takao@nims.go.jp (T.M.)

[2] Graduate School of Pure and Applied Sciences, University of Tsukuba, 1-1-1 Tennodai, Tsukuba, Ibaraki, 305-8577, Japan; sakurai.takeaki.ft@u.tsukuba.ac.jp (T.S.)

* Correspondence: MORI.Takao@nims.go.jp (T.M.); PANG.Hong@nims.go.jp (H.P.); sakurai.takeaki.ft@u.tsukuba.ac.jp (T.S.)

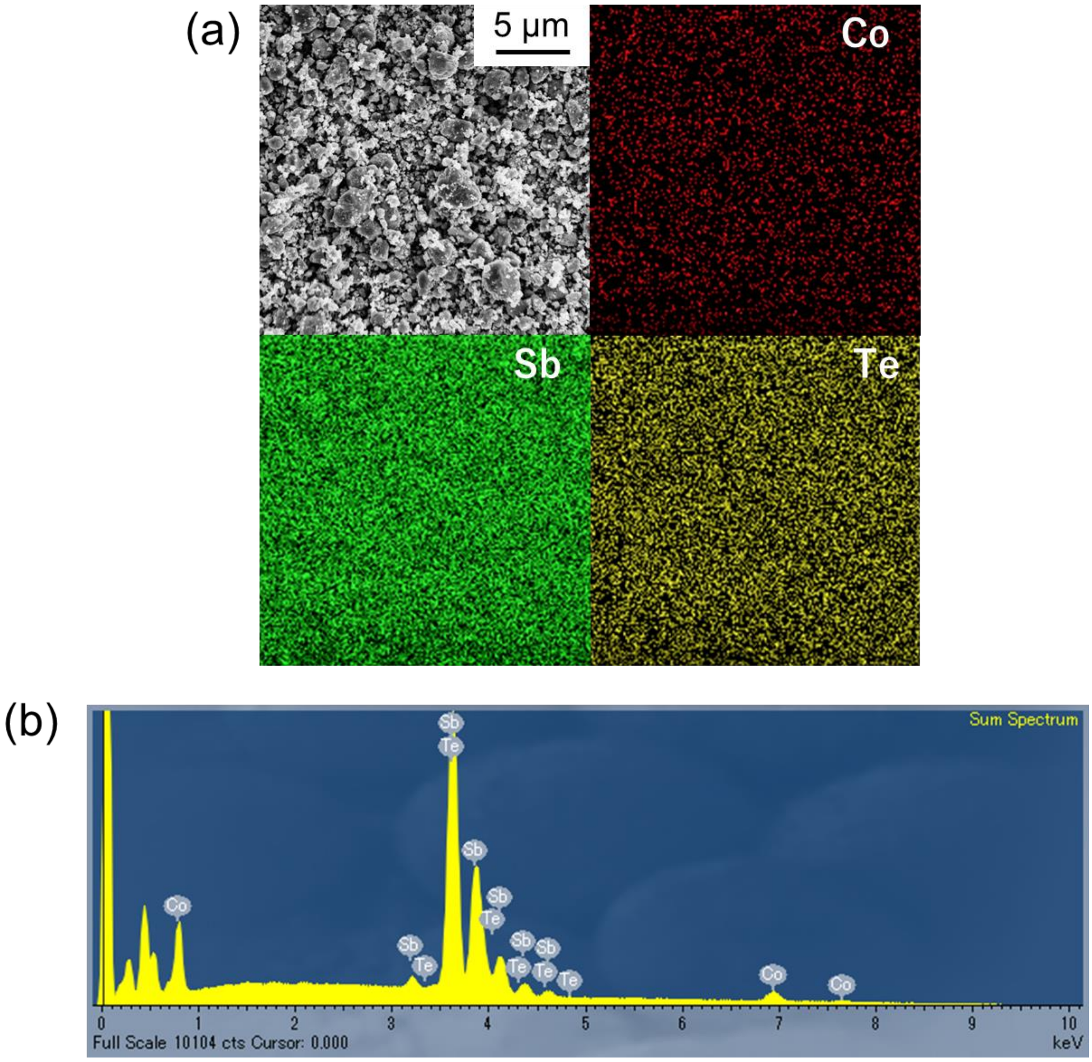

Figure S1. (a) SEM image and EDX mapping images of the ball-milled $CoSb_{2.95}Te_{0.05}$ powder; (b) the corresponding EDX spectrum.

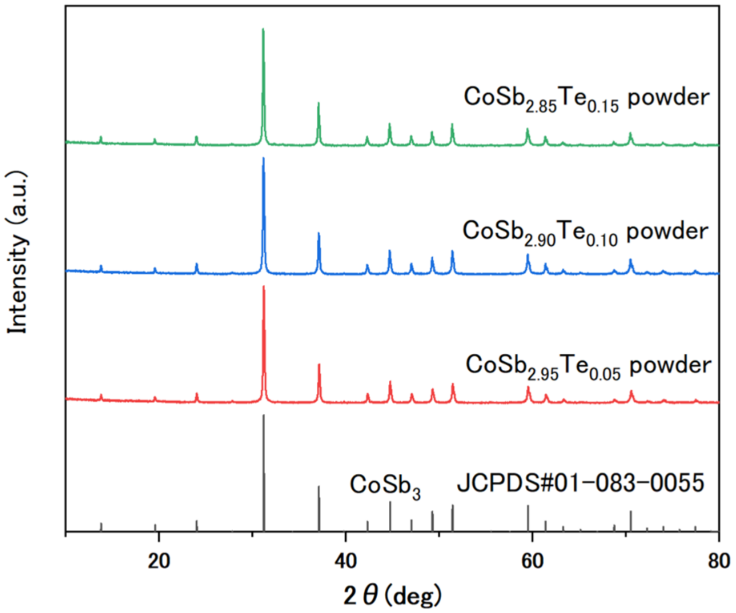

Figure S2. XRD patterns of the ball-milled $CoSb_{3-x}Te_x$ powder with different amount of Te dopants (x = 0.05, 0.10, 0.15).

Table S1. Cell parameters and reliability factors obtained from Rietveld refinement of XRD patterns (λ_{Cu} = 1.54056 Å) of the $CoSb_{3-x}Te_x$ ball milled powders and $XCoSb_{2.95}Te_{0.05}$ / 1-X PEDOT:PSS hybrid films

$CoSb_{3-x}Te_x$ ball milled powders					
sample	a (Å)	V (Å^3)	R_{Bragg}	R_F	Chi^2
x=0.15	9.0501	741.2	4.13	4.13	1.48
x=0.10	9.0487	740.9	4.82	4.2	1.49
x=0.05	9.0413	739.1	4.27	4.27	1.44
$XCoSb_{2.95}Te_{0.05}$ / 1-X PEDOT:PSS films					
X = 20%	9.0405	738.9	4.45	4.91	1.20
X = 40%	9.0367	737.9	3.43	3.58	1.38
X = 60%	9.0367	737.9	3.81	4.3	1.39
X = 80%	9.0358	737.7	5.96	5.07	1.58
X = 95%	9.0368	738.0	5.88	4.72	1.75
X = 97%	9.0401	738.1	3.65	3.07	1.54
X = 98%	9.0375	738.1	8.24	6.08	2.03

Table S2. Thermoelectric properties of PEDOT:PSS with 5 vol% of DMSO and the $CoSb_{2.95}Te_{0.05}$ skutterudite film sample at room temperature.

	Seebeck coefficient [μV/K]	Electrical conductivity [S/cm]	Power factor [μW/m K^2]	Thermal conductivity [W/m K]
PEDOT:PSS (DMSO 5vol%)	16.8	1195	33.8	0.27 [1]
$CoSb_{2.95}Te_{0.05}$ powder film	-174.8	0.031	0.096	7.33*

* The thermal conductivity of the $CoSb_{2.95}Te_{0.05}$ powder film is substituted by the thermal conductivity of the $CoSb_{2.95}Te_{0.05}$ bulk sample to plot the series-connected model in Figure 3.

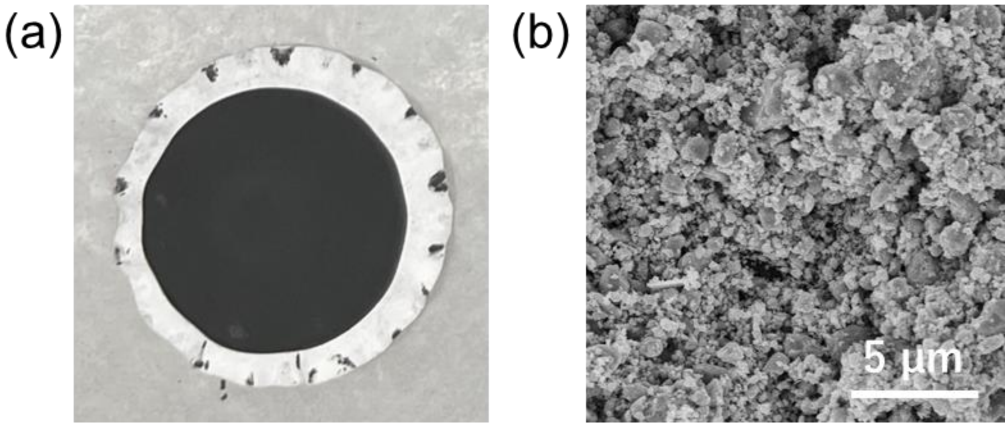

Figure S3. Cold-pressed $CoSb_{2.95}Te_{0.05}$ powder film: (a) digital photo image; (b) SEM image.

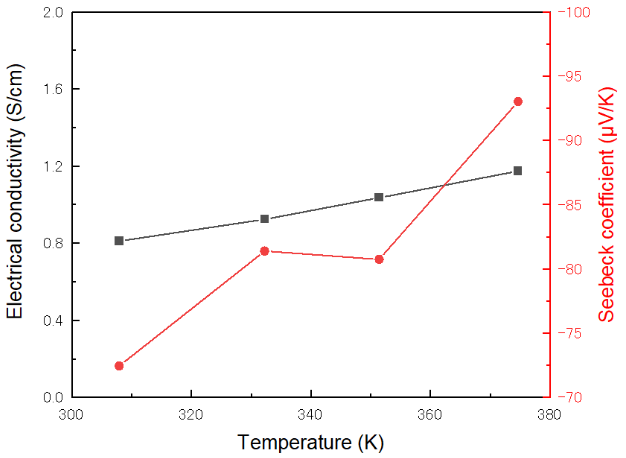

Figure S4. Electrical conductivity and the Seebeck coefficient of the 98wt% $CoSb_{2.95}Te_{0.05}$ hybrid film with increasing temperature

Table S3. Thermoelectric properties of the $CoSb_{3-x}Te_x$ (x = 0.05, 0.10, 0.15) bulk sample at room temperature.

	Seebeck coefficient [μV/K]	Electrical conductivity [S/cm]	Power factor [μW/m K^2]
x=0.05	-179	1050	3350
x=0.10	-139	1360	2620
x=0.15	-124	1550	2370

References

1. Li, Z.; Sun, H.; Hsiao, C.; Yao, Y.; Xiao, Y.; Shahi, M.; Jin, Y.; Cruce, A.; Liu, X.; Jiang, Y.; et al. A Free-Standing High-Output Power Density Thermoelectric Device Based on Structure-Ordered PEDOT:PSS. *Adv. Electron. Mater.* **2018**, *4*, 1–8.
